# PLANS4CARE: TRANSLATING EVIDENCE FROM IN-PERSON CAREGIVER INTERVENTIONS TO A DIGITAL PLATFORM

**DOI:** 10.1093/geroni/igae098.2040

**Published:** 2024-12-31

**Authors:** Eric Jutkowitz, Catherine Piersol, Sokha Koeuth, Laura Gitlin

**Affiliations:** Brown University, Brown University, Rhode Island, United States; Jefferson College of Rehabilitation Sciences, Philadelphia, Pennsylvania, United States; Plans4. Care, Bala Cynwyd, Pennsylvania, United States; Drexel University, Philadelphia, Pennsylvania, United States

## Abstract

Our research group has developed and/or evaluated numerous evidence-based non-drug dementia caregiver interventions (e.g., Tailored Activity Program and Skills2Care). For these interventions, healthcare professionals conduct in-home assessments to evaluate cognitive function of the person living with dementia, determine the caregiver’s care challenges (e.g., behaviors), and provide tailored strategies. In randomized trials, these interventions improved caregiver outcomes (e.g., less time caregiving), person with dementia outcomes (e.g., reduced behavioral symptoms), and health system outcomes (e.g., cost savings). Informed by this evidence, our team engaged in an iterative process to create a digital platform that caregivers can use on-demand. First, we mined the clinical trial data and identified 80 caregiver challenges that we grouped into 8 domains: for persons with dementia, behaviors, self-care mobility, household tasks, leisure participation, and healthcare; caregiver-centered challenges (need for respite), and general dementia care practices. Second, we reviewed unique strategies documented and published, which we grouped into 6 categories: communication, cueing, simplifying activity, enhancing, engagement, modifying environment, and taking care of self. Third, two healthcare providers on our team independently mapped care challenges to evidence-based strategies based on the level of cognitive function. Our approach generated a database of 80 care challenges linked to >1500 evidence-based strategies classified by six levels of cognitive functioning (high to low), and a simple user interface for caregivers to generate personalized care plans. Translating in-person sessions to digital platforms and maintaining tailoring and clinical relevance requires an iterative developmental process that is data-driven and informed by caregiver input.

